# Gingival Recession After Combined Orthodontic–Orthognathic Treatment: A Systematic Review of Clinical Studies with Emphasis on Mandibular Incisors and Adjunctive Periodontal Therapies

**DOI:** 10.3390/jcm15051793

**Published:** 2026-02-27

**Authors:** Alexandru Mester, Gabriel Armencea, Andrei Tent, Dacian Sabau, Florin Gligor Onisor, Simion Bran

**Affiliations:** 1 Department of Oral Health, University of Medicine and Pharmacy “Iuliu Hatieganu”, 400012 Cluj-Napoca, Romania; 2 Department of Maxillofacial Surgery and Implantology, University of Medicine and Pharmacy “Iuliu Hatieganu”, 400012 Cluj-Napoca, Romania; 3 Department of Oral and Maxillo-Facial Surgery, Faculty of Medicine and Pharmacy, University of Oradea, 410087 Oradea, Romania

**Keywords:** gingival recession, periodontal recession, orthodontic–orthognathic treatment, Le Fort I osteotomy, sagittal split osteotomy

## Abstract

**Background and Objectives:** Combined orthodontic–orthognathic treatment is the standard approach for managing severe dentofacial deformities. Nevertheless, its potential impact on periodontal tissues, particularly gingival recession (GR), remains a matter of clinical concern. This study aimed to evaluate the relationship between orthodontic–orthognathic procedures and GR based on available clinical evidence, with particular focus on potential risk modifiers, including orthodontic mechanics, surgical factors, and periodontal phenotype. **Materials and Methods:** This systematic review was conducted in accordance with the Preferred Reporting Items for Systematic Reviews and Meta-Analyses (PRISMA) 2020 guidelines. The review question was formulated using the PICO framework. **Results:** Seven observational clinical studies were included, with sample sizes ranging from 24 to 40 patients. GR outcomes varied considerably according to surgical procedure, orthodontic mechanics, adjunctive therapies, and patient-specific factors. Overall certainty of evidence was low–moderate. **Conclusions:** GR following combined orthodontic–orthognathic treatment is inconsistently reported and influenced by multiple clinical variables. Evidence suggesting roles for periodontal phenotype and adjunctive therapies, including PAOO, remains limited and context-dependent. Overall certainty of evidence is low–moderate, and findings should be interpreted cautiously.

## 1. Introduction

Severe dentomaxillary and skeletal anomalies represent a unique category of pathology in oral and maxillofacial surgery, as their management is complex and extends over a prolonged period of time [1]. Owing to disorders of maxillary and mandibular bone development in relation to the cranial base, an exclusively orthodontic therapeutic approach carries a high risk of failure [2]. In such complex cases, orthognathic surgery is a mandatory and distinct stage in patient management [3]. Surgical procedures are generally performed at the completion of craniofacial growth and are preceded and followed by orthodontic treatment [4]. Orthognathic surgery may range from relatively simple procedures, such as surgically assisted rapid palatal expansion (SARPE), to more complex bimaxillary interventions involving controlled maxillary (Le Fort I) and mandibular (bilateral sagittal split osteotomy—BSSO) osteotomies. These procedures allow for advancement, retrusion, rotation, impaction, or disimpaction of the jaws, with the aim of restoring craniofacial symmetry, functional balance, and aesthetic harmony [5].

Failure to address this type of pathology in a multidisciplinary manner, or incorrect timing in the orthodontic–orthognathic approach, can lead to the development of major functional and aesthetic disorders such as facial asymmetry, periodontal disease with gingival recession (GR), temporomandibular joint disorders (TMDs), facial pain, and chewing, speech, or swallowing disorders in severe cases [6]. Within this clinical picture, preoperatively present GR has a major impact on facial aesthetics [7]. Uneven transmission of masticatory forces in patients with severe malocclusions or improperly conducted orthodontic treatments can lead to pathological bone resorption with secondary GR and exposure of the tooth roots [1,2,3,4]. This may result in dental hypersensitivity of varying degrees, an increased risk of carious lesions, or dental mobility in severe cases [7,8]. Poor oral hygiene in these patients can further aggravate these symptoms, leading to faster disease progression and irreversible complications [9].

Orthodontic–orthognathic treatment aims to establish craniofacial skeletal harmony by achieving improved facial balance, proportions, and functional occlusion, with a normal condyle–centric relation (CR) [2]. These corrections also aim to improve the patient’s periodontal health by halting the progression of GR or even preventing its occurrence in cases where it is not yet present [7,8,9]. A major concern, however, is the development of GR secondary to surgical interventions when it was not previously present [1,2,3,4,5,6]. This outcome has been reported by several authors, all of whom consider it a sign of significant treatment failure [1,2,3,4,5,6]. Postoperative GR may be caused by multiple factors, including intraoperative surgical trauma to the periodontium, the patient’s gingival phenotype, improperly conducted orthodontic treatment, or a combination of these factors [1,2,3,4,5,6]. Randomized clinical data demonstrate that PAOO can influence periodontal indices and alveolar support during accelerated orthodontic treatment, providing a clinical context for its potential protective role in combined orthodontic–orthognathic pathways [7]. However, there is currently no consensus in the literature regarding the primary causes of postoperative GR, nor are there clear recommendations for its prevention, as authors’ opinions remain divided [7,8,9].

In light of these limitations, this systematic review aims to evaluate the available clinical evidence on GR following combined orthodontic–orthognathic treatment, with particular focus on potential risk modifiers, including orthodontic mechanics, surgical factors, and periodontal phenotype.

## 2. Materials and Methods

### 2.1. Study Design and Protocol Registration

This study was designed as a systematic review without meta-analysis, conducted in accordance with the Preferred Reporting Items for Systematic Reviews and Meta-Analyses (PRISMA) 2020 guidelines. A quantitative meta-analysis was not performed because of substantial heterogeneity among the included clinical studies in terms of study design, outcome definitions, recession measurement methods, and follow-up duration.

Prospective protocol registration (e.g., PROSPERO) was not undertaken. Although recommended, registration is not mandatory for systematic reviews and is primarily intended to minimize reporting bias and duplication, particularly in reviews planning quantitative synthesis. The present review followed a predefined methodological framework specifying the research question, eligibility criteria, study selection, and data extraction procedures, ensuring transparency and reproducibility.

### 2.2. Focused Research Question

The focused research question was “What is the presence and extent of gingival recession following combined orthodontic–orthognathic treatment compared with baseline or control conditions?”

### 2.3. Eligibility Criteria

- Studies were considered eligible if they met the following criteria:
- Population: Human subjects undergoing combined orthodontic and orthognathic treatment.
- Intervention: Any orthodontic–orthognathic surgical approach (single or bimaxillary).
- Outcome: Presence, prevalence, or severity of GR measured clinically. GR was defined as apical displacement of the gingival margin relative to the cemento-enamel junction with clinical exposure of the root surface. Studies were eligible if recession was reported as a measurable clinical parameter (e.g., ≥1 mm recession depth or continuous measurements in millimeters) assessed using periodontal probing or standardized clinical indices.
- Study design: Randomized controlled trials, non-randomized clinical trials, prospective or retrospective cohort studies, and case–control studies.
- Language: Articles published in English.
- Publication status: Full-text articles available.

Studies reporting mixed periodontal outcomes (e.g., gingival thickness, attachment level, mucogingival parameters) were included only when GR outcomes were presented separately or could be extracted independently. Studies lacking recession-specific data were excluded.

The following studies were excluded: case reports, case series with fewer than 10 patients, reviews, editorials, letters, and conference abstracts; animal or in vitro studies; studies evaluating orthodontic treatment alone without orthognathic surgery; studies not reporting GR as an outcome; and studies with insufficient or unclear outcome data.

### 2.4. Information Sources and Search Strategy

A comprehensive electronic literature search was conducted in the following databases: PubMed/MEDLINE, Web of Science, and Cochrane Library. The search covered all available records from database inception to the most recent update prior to manuscript preparation. A combination of Medical Subject Headings (MeSH) terms and free-text keywords was used (Table S1). The search strategy was adapted for each database. The following keywords were used: gingival recession, periodontal recession, gingival margin, orthognathic surgery, LeFort I osteotomy, maxillary osteotomy, mandibular osteotomy, jaw surgery, and sagittal split osteotomy. Additionally, the reference lists of included studies and relevant reviews were manually screened to identify further eligible articles 

### 2.5. Study Selection

All identified records were imported into reference management software, and duplicates were removed. Two independent reviewers screened titles and abstracts for eligibility. Full-text articles were then assessed independently for inclusion. Any disagreements were resolved through discussion or consultation with a third reviewer. The study selection process was documented using a PRISMA flow diagram and checklist (File S1).

### 2.6. Data Extraction

Data were independently extracted by two reviewers using a standardized data extraction form. The following information was collected: author(s) and year of publication, study design, sample size and demographic characteristics, type of orthodontic–orthognathic treatment, duration of follow-up, methods used to assess GR, main outcomes related to GR, confounding factors, and limitations. Any discrepancies in data extraction were resolved by consensus (Table S2).

When relevant methodological or outcome data were missing or unclear in the included studies, attempts were made to contact the corresponding authors for clarification. No statistical imputation was performed. If information remained unavailable, variables were recorded as ‘not reported,’ and studies were retained, provided GR outcomes could be clearly extracted. Incomplete reporting was considered a potential source of bias during interpretation.

### 2.7. Risk of Bias Assessment

The methodological quality and risk of bias of the included studies were assessed independently by two reviewers. For observational studies, the Newcastle–Ottawa Scale (NOS) was applied, evaluating three domains: Selection, Comparability, and Outcome. Item-level judgments were recorded for each domain, and total scores were used to categorize studies as low, moderate, or high risk of bias (Table S3).

### 2.8. Data Synthesis

Due to substantial heterogeneity in study design, follow-up duration, recession measurement methods, baseline periodontal status, orthodontic mechanics, and adjunctive periodontal therapies, quantitative meta-analysis was not appropriate. Therefore, a structured qualitative synthesis was performed, including subgroup narrative comparisons based on adjunctive therapies (e.g., PAOO vs. non-PAOO) and follow-up duration (short- vs. long-term). When sufficient homogeneous data were available, quantitative analysis was considered. Results are summarized descriptively and presented in tables.

## 3. Results

### 3.1. Study Selection

**Figure 1:**
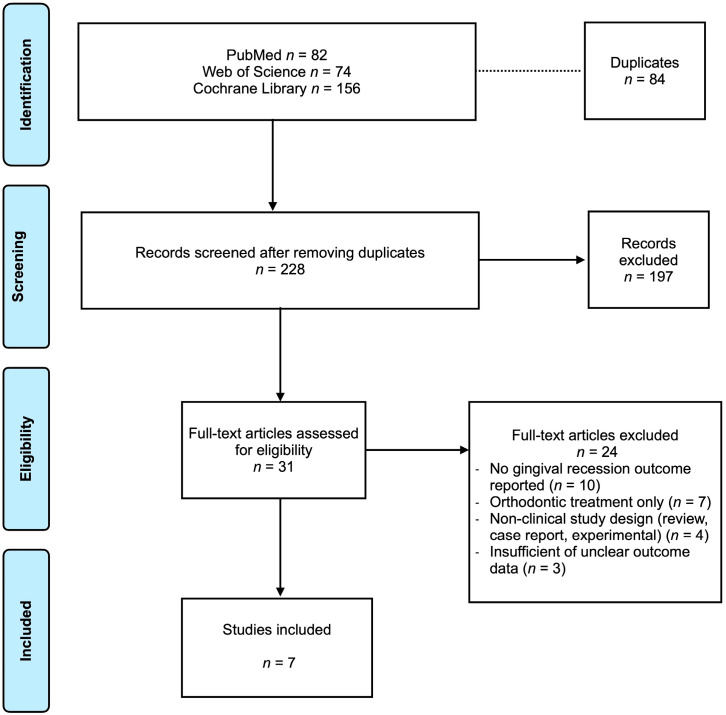
Prisma flowchart.

The literature search across PubMed/MEDLINE, Web of Science, and the Cochrane Library yielded 312 records. After the removal of 84 duplicates, 228 articles underwent title and abstract screening. 197 records were excluded because they did not address combined orthodontic–orthognathic treatment with clinical GR outcomes or were ineligible study designs (e.g., case reports, reviews). Overall, 31 full-text articles were assessed for eligibility; 24 were excluded owing to lack of recession data, absence of combined treatment, or inadequate outcome reporting. Ultimately, 7 clinical studies met all inclusion criteria and were included in this systematic review (Figure 1).

### 3.2. Characteristics of Included Studies

The characteristics of the included studies are summarized in Table 1. All seven investigations were observational clinical studies evaluating gingival or mucogingival outcomes in patients undergoing combined orthodontic–orthognathic treatment. Sample sizes ranged from 24 to 40 patients, with outcomes assessed at the level of individual teeth or tooth regions. Follow-up periods varied from immediate postoperative evaluation to long-term observation, and outcome measures primarily consisted of clinical assessments of GR and periodontal parameters. Incomplete reporting, including data unavailable after author contact, limited quantitative comparability and further supported the decision to perform qualitative synthesis.

Liu et al. (2024) conducted a retrospective cohort study in skeletal Class III patients undergoing bimaxillary orthognathic surgery, with or without adjunctive periodontally accelerated osteogenic orthodontics (PAOO) [10]. The authors assessed gingival thickness, keratinized gingiva width, and GR at the mandibular incisors during post-treatment follow-up of up to 12 months.

Saab et al. (2023) performed a comparative observational study evaluating GR of mandibular incisors immediately after treatment in Class III patients managed with either compensatory orthodontic treatment or combined orthodontic–orthognathic surgery [11].

Two prospective clinical studies by Weinspach et al. (2011) evaluated the short-term periodontal effects of orthognathic surgery, including bilateral sagittal split osteotomy with or without Le Fort I osteotomy [12,13]. Both studies reported quantitative periodontal changes, with particular emphasis on early postoperative buccal GR assessed at 6 weeks.

Ari-Demirkaya et al. (2008) conducted a case–control study examining periodontal changes in the mandibular incisor region following mandibular setback surgery, focusing on the influence of postoperative relapse forces during a follow-up period of 6 to 12 months [14].

Carroll et al. (1992) performed a comparative clinical study assessing long-term periodontal outcomes in patients treated with orthodontics alone versus those receiving combined orthodontic and Le Fort I orthognathic surgery, with follow-up ranging from 1 to 10 years [15].

Finally, Foushee et al. (1985) conducted an observational clinical study evaluating periodontal parameters in the mandibular anterior region following orthognathic surgery, reporting the prevalence of clinically significant GR during a 6- to 12-month post-treatment period [16].

### 3.3. Gingival Recession Following Combined Treatment

The included studies demonstrated variability in several methodological and clinical parameters, including study design, follow-up duration, recession measurement approach, baseline periodontal status, orthodontic decompensation mechanics, and the use of adjunctive periodontal therapies. These differences contributed to outcome variability and precluded quantitative pooling. A structured overview of heterogeneity is presented in Table 2.

#### Overall Recession Outcomes

The evidence regarding GR following combined orthodontic–orthognathic treatment is heterogeneous. Liu et al. (2024) found a significant increase in GR in the mandibular incisor region among patients without PAOO adjunctive therapy [10]. In the non-PAOO (NS) group, GR increased by 47.62%, with the odds of recession after orthodontic-orthognathic treatment being 14.77 times higher than in the PAOO group (*p* < 0.05). Gingival thickness and keratinized gingiva width also decreased significantly in the NS group but increased in the PAOO group. In contrast, Saab et al. (2023) reported no statistically significant difference in GR of mandibular incisors when comparing immediate postsurgical outcomes with compensatory orthodontic treatment alone, suggesting that the orthognathic surgical component per se did not independently increase recession in their cohort [11].

Weinspach et al. (2011) observed significant increases in buccal GR from 0.10 ± 0.16 mm at baseline to 0.31 ± 0.31 mm at six weeks after surgery (*p* < 0.001), indicating short-term recession changes following surgery [12]. Also, Weinspach et al. (2011) identified no significant microbiological changes [13]. Between one and six weeks postop, PPD increased on oral sites and GR on buccal sites, reaching statistical significance (*p* = 0.013 at 1 week; *p* = 0.001 at 6 weeks). In the incision area, the development of GR was significantly higher on the test (buccal) than on the control sites (oral).

Ari-Demirkaya et al. (2008) indicated that decompensation orthodontic movement prior to mandibular setback did not significantly worsen periodontal structures in the short term, and early postoperative relapse forces did not exhibit additional adverse periodontal effects, implying that orthodontic biomechanics may have a larger impact than surgical movements alone [14]. Carroll et al. (1992) identified no significant long-term differences in periodontal status, including GR, when comparing patients treated with orthodontics alone versus those with combined orthognathic therapy, although minor changes in probing depths and attachment level occurred in specific osteotomy subgroups [15]. Foushee et al. (1985) reported that a statistically significant decrease in keratinized and attached gingiva occurred after orthognathic therapy in the mandibular anterior region, and clinically significant recession was present in 6 of 24 patients [16].

### 3.4. Mandibular Incisor Region

Six of the included studies directly assessed mandibular incisor gingival outcomes (Table 3). Taken together, these studies demonstrate that mandibular incisor recession may occur following combined treatment, particularly where the periodontal phenotype is thin or adjunctive periodontal procedures are not performed, but the contribution of the surgical component remains inconsistent across studies. Heterogeneity within this subgroup was primarily driven by differences in orthodontic decompensation protocols, periodontal phenotype distribution, and duration of follow-up.

### 3.5. Additional Influencing Factors

Several studies identified clinical factors that may influence GR outcomes. Periodontal phenotype variables, such as gingival thickness and alveolar bone dimensions, were significantly associated with recession risk. Multivariate regression in Liu et al. (2024) showed that thin gingiva (<0.72 mm), alveolar bone height > 2.36 mm, and bone thickness < 0.45 mm were significant predictors of postoperative recession [10]. Orthodontic decompensation (inclination) magnitude and preoperative periodontal conditions in studies examining relapse forces contributed to periodontal variability but did not uniformly predict recession outcomes [10,11,12,13,14,15,16].

### 3.6. Risk of Bias and Evidence Certainty

Using the Newcastle–Ottawa Scale (NOS) (Table 4) for observational studies, the methodological quality ranged from moderate to high across included studies, with concerns primarily related to retrospective designs, variability in follow-up durations, and lack of standardized recession measurement methods. Overall certainty of evidence remains low to moderate due to these limitations.

### 3.7. Data Synthesis

Due to considerable heterogeneity in outcome definitions, measurement techniques, and follow-up intervals, quantitative meta-analysis was not possible. Therefore, a qualitative synthesis and direction of effect summary were conducted (Table 5). The influence of risk of bias on the synthesis was considered by prioritizing findings from studies with lower risk, interpreting results from higher-risk studies cautiously, and avoiding quantitative pooling where methodological heterogeneity or bias could compromise validity.

Across the seven included studies, the presence and extent of GR following combined orthodontic–orthognathic treatment varied according to surgical procedure, adjunctive therapy, and patient-specific factors. Liu et al. (2024) [10] reported that patients undergoing orthodontic–orthognathic treatment without PAOO experienced significantly higher mandibular incisor recession compared with those who received PAOO, who exhibited minimal recession. Saab et al. (2023) [11] found no significant difference in recession between patients who received compensatory orthodontic treatment versus those who underwent surgical treatment, suggesting that surgery per se did not independently increase recession. Weinspach et al. (2011) [12,13] observed small but statistically significant increases in buccal GR following BSSO and/or Le Fort I surgery (from 0.10 ± 0.16 mm at baseline to 0.31 ± 0.31 mm postoperatively).

Long-term outcomes from the study by Carroll et al. (1992) [15] indicated no significant increase in GR over 1–10 years, although minor localized effects were noted. Similarly, in the study by Foushee et al. (1985) [16], the authors reported clinically significant recession in 6 out of 24 patients, primarily affecting the labial surfaces. In contrast, Ari-Demirkaya et al. (2008) [14] demonstrated that mandibular setback combined with orthodontic decompensation did not significantly influence incisor recession, highlighting the potential role of orthodontic mechanics rather than surgical movements. Finally, the study by Weinspach et al. [12,13] found small increases in GR on labial surfaces in the early postoperative period, suggesting a transient effect associated with surgery.

## 4. Discussion

The present review evaluated the presence and extent of GR following combined orthodontic–orthognathic treatment. The overall findings indicate that although GR may occur—particularly in the mandibular incisor region—it is not a uniform or inevitable outcome of orthognathic surgery. Instead, recession appears to be influenced by a multifactorial interaction between periodontal phenotype, orthodontic biomechanics, presurgical decompensation, and adjunctive periodontal measures, rather than by the surgical procedure itself [1,3,4].

### 4.1. Is the Orthodontic–Orthognathic Approach a True Risk Factor?

The question of whether combined orthodontic–orthognathic treatment represents a major risk factor for GR remains controversial. The systematic review by Mota de Paulo et al. [3] reported that GR may be observed following combined treatment. The authors emphasized the heterogeneity of study designs and outcomes and concluded that the available evidence does not allow surgery to be isolated as an independent etiological factor [3]. This conclusion aligns with the findings of Carroll et al. [15] and Ari-Demirkaya et al. [14], both of whom found no clinically significant long-term periodontal deterioration attributable to orthognathic surgery.

This interpretation is further supported by the evidence-based review by Al-Jewair [17], who concluded that orthodontic–orthognathic surgical treatment may not be a major risk factor for GR when appropriate orthodontic control, periodontal assessment, and oral hygiene are ensured. Collectively, these data suggest that the recession observed after combined treatment is more likely related to patient-specific and orthodontic variables rather than to orthognathic surgery per se [17].

### 4.2. Orthodontic Decompensation and Periodontal Limits

A key finding emerging from both the included studies and the external literature is the role of orthodontic decompensation (in particular, mandibular incisor repositioning) as a critical determinant of GR risk. Excessive labiolingual movement beyond the alveolar envelope may predispose patients to alveolar bone dehiscence and subsequent recession, especially in individuals with a thin periodontal phenotype [3,18].

The study by Demirsoy et al. [18] addressed this issue, demonstrating the development of periodontal bone defects during presurgical orthodontic decompensation in Class III double-jaw surgery patients. These findings support the concept that periodontal compromise may develop before surgery, thereby increasing vulnerability to postoperative recession. This observation is in line with the results of Liu et al. [10], who identified gingival thickness and alveolar bone dimensions as strong predictors of postoperative recession and demonstrated a protective effect of PAOO in high-risk patients. Randomized clinical evidence indicates that PAOO may positively influence periodontal indices and alveolar support during accelerated orthodontic treatment, supporting its potential role as a protective adjunct in combined orthodontic–orthognathic therapy [7].

### 4.3. Periodontally Compromised Patients and Multidisciplinary Care

The feasibility of orthodontic–orthognathic treatment in patients with compromised periodontal conditions has also been explored in the literature. Halimi and Zaoui [19] reported successful surgical–orthodontic management of patients with severe periodontal disorders, emphasizing the importance of strict periodontal control, conservative orthodontic mechanics, and interdisciplinary coordination. Although limited by its case-based nature, this study reinforces the notion that periodontal vulnerability does not represent an absolute contraindication to combined treatment when managed appropriately [19,20,21].

Similarly, the case report by Liu et al. [2] highlighted the benefits of integrating periodontal phenotype modification into the orthognathic treatment pathway. Their multidisciplinary approach demonstrated improved periodontal stability and reduced risk of recession, supporting current consensus recommendations advocating for periodontal phenotype assessment and modification in high-risk patients [8]. Systematic evidence supports the efficacy of injectable platelet-rich fibrin for gingival phenotype modification, which may be considered alongside PAOO when planning periodontal risk mitigation in orthognathic patients [22].

### 4.4. Surgical Technique and Segmental Osteotomies

More recent studies have demonstrated that orthognathic procedures such as bilateral sagittal split osteotomy and Le Fort I osteotomy may indirectly influence gingival margins by modifying soft tissue tension, muscular attachments, and local vascular supply [21]. Although these changes are generally transient, they may increase susceptibility to marginal tissue displacement in patients with thin gingival biotypes or reduced alveolar thickness [21].

Concerns have been raised regarding the potential periodontal impact of more complex surgical techniques, such as segmental maxillary osteotomies performed in conjunction with bimaxillary surgery. However, available evidence suggests that these procedures are generally safe when proper surgical principles are applied [12,13,20,21].

Case-based and cohort analyses further suggest that mandibular incisors represent a particularly vulnerable region following orthodontic–orthognathic treatment [12,13,20,21]. Retrospective data indicate that GR in this area is more closely associated with postoperative orthodontic tooth positioning and periodontal phenotype than with the surgical procedure itself [2]. This supports the concept that orthognathic surgery acts as a modifying factor rather than a primary etiological agent.

Posnick et al. [20] reported favorable outcomes and acceptable complication rates for segmental maxillary osteotomies, without identifying GR as a frequent or severe adverse outcome. These findings support the view that surgical complexity alone does not necessarily translate into periodontal deterioration.

Short-term studies, such as those by Weinspach et al. [12,13], have documented small but statistically significant increases in buccal GR in the early postoperative period. These changes are likely related to transient surgical trauma and healing dynamics rather than permanent periodontal damage, particularly in the absence of long-term follow-up demonstrating progression. Overall, current evidence indicates that GR following orthodontic–orthognathic treatment is primarily influenced by individual periodontal susceptibility and treatment mechanics [12,13,19,20,21].

According to Liu et al. [2] and Weinspach et al. [12,13], orthognathic surgery appears to be associated with GR in some clinical contexts; however, current observational evidence does not allow causal relationships to be established. The recession is procedure-related rather than disease-driven, predominantly affects buccal sites, and is highly dependent on pre-existing periodontal phenotype. Importantly, periodontal augmentation strategies such as PAOO can significantly mitigate this risk, transforming orthognathic surgery from a periodontal risk factor into a controllable variable.

Orthognathic surgery itself does not appear to be an independent risk factor but may exacerbate existing vulnerabilities when periodontal limits are exceeded [12,13,19,20,21].

### 4.5. Reconciling Conflicting Evidence

The variability in GR outcomes across studies can be explained by structured sources of heterogeneity, including study design, follow-up duration, recession measurement methods, baseline periodontal status, orthodontic mechanics, and adjunctive periodontal therapies [3]. Subgroup narrative synthesis indicates that adjunctive periodontal approaches (e.g., PAOO) and longer follow-up periods are associated with more stable periodontal outcomes, whereas short-term studies tend to report transient recession changes [10,11,12,13,14,15,16].

Overall, the evidence suggests that GR following combined orthodontic–orthognathic treatment should not be regarded as an unavoidable complication [3,17]. Instead, it should be considered a potentially preventable outcome through careful case selection, controlled orthodontic mechanics, periodontal phenotype assessment, and interdisciplinary treatment planning [3,17].

### 4.6. Strengths and Limitations

This systematic review followed PRISMA guidelines and applied strict inclusion criteria, focusing on clinical studies evaluating gingival recession (GR) after combined orthodontic–orthognathic treatment. The structured risk-of-bias assessment and qualitative synthesis enabled critical interpretation of both short- and long-term periodontal outcomes, while the inclusion of both classical and contemporary studies provided a broad clinical perspective.

The main limitation is the low-to-moderate certainty of the evidence, as all included studies were observational. Substantial heterogeneity in study design, outcome definitions, follow-up duration, and measurement methods precluded meta-analysis. Important modifying factors—such as orthodontic mechanics, periodontal phenotype, oral hygiene, and baseline periodontal status—were inconsistently reported, and in some studies, baseline periodontal conditions were not described, limiting comparability and causal interpretation. Additional limitations include potential publication bias and restriction to English-language studies. Overall, findings should be interpreted as associations rather than causal relationships, with low–moderate certainty of evidence.

### 4.7. Clinical Implications

Periodontal risk assessment prior to treatment is recommended for all orthodontic–orthognathic patients, particularly in the mandibular incisor region [23,24]. In patients with a thin periodontal phenotype or limited keratinized tissue, clinicians should consider phenotype modification and careful control of orthodontic decompensation [25,26]. Adjunctive approaches such as PAOO may be considered in selected high-risk cases; however, supporting evidence is limited and observational [27,28]. Interdisciplinary planning and regular periodontal monitoring are essential for the early detection and management of gingival recession [23,24].

## 5. Conclusions

Gingival recession following combined orthodontic–orthognathic treatment is associated primarily with periodontal phenotype and orthodontic tooth movement, particularly in the mandibular incisor region, based on observational evidence of low–moderate certainty. A causal relationship cannot be established. Further well-designed prospective and randomized studies are required to clarify causality and quantify the independent contribution of surgical and orthodontic factors.

## Figures and Tables

**Table 1:** Characteristics of included studies.

Study	Study Design	Sample Size	Type of Orthognathic Surgery	Orthodontic Phase Evaluated	Follow-Up Duration	Tooth Region Assessed	Baseline Periodontal Status Reported	Recession Measurement Method	Examiner Calibration	Definition of GR	Baseline Periodontal Therapy	Main GR Outcome	Statistical Reporting (Effect Size/*p*-Value/CI)	Reported Periodontal Parameter Values (from Original Studies)
Liu et al., 2024 [10]	Retrospective cohort	33 patients	Orthognathic ± PAOO	Post-treatment	Up to 12 months	Mandibular incisors	Yes	Clinical periodontal exam	Not reported	CEJ–gingival margin	Supportive therapy	Higher GR in non-PAOO group	Significant between-group difference; CI not reported	GR depth values reported per mandibular incisor group; gingival thickness and keratinized tissue measured quantitatively
Saab et al., 2023 [11]	Comparative observational	40 patients	Surgical vs. compensatory orthodontics	Immediate	Immediate	Mandibular incisors	Not reported	Photographic measurement	Not reported	CEJ–gingival margin	Not reported	No difference between groups	*p*-values reported; CI not reported	GR measured from CEJ to the gingival margin pre- and post-treatment for both groups (mm values reported)
Weinspach et al., 2011 [12]	Prospective clinical	15 patients	Le Fort I/BSSO	Early postoperative	1–6 weeks	Buccal/oral	Yes	Periodontal probing	Not reported	CEJ–gingival margin change	Not reported	Increase in buccal GR	*p* = 0.013 (1 week), *p* = 0.001 (6 weeks); CI not reported	GR increased from 0.10 ± 0.16 mm (baseline) to 0.21 ± 0.23 mm (1 week) and 0.31 ± 0.31 mm (6 weeks); PPD and CAL also recorded
Weinspach et al., 2011 [13]	Prospective clinical	15 patients	Single or bimaxillary surgery	Early postoperative	1–6 weeks	Buccal/oral	Yes	Periodontal probing	Not reported	Clinical GR measurement	Not reported	Increased GR on buccal sites	Significant GR changes; CI not reported	GR, PPD, CAL, BOP and plaque index measured pre-op and post-op; higher GR on buccal sites
Ari-Demirkaya et al., 2008 [14]	Case–control	36 patients	Mandibular setback	Post-treatment	~7 months	Mandibular incisors	Yes	Clinical + radiographic	Not reported	Periodontal mucogingival parameters	Not reported	No significant GR effect	No significant association; CI not reported	Sulcus depth, alveolar height, and mucogingival parameters recorded pre- and post-surgery; no significant GR increase
Carroll et al., 1992 [15]	Comparative clinical	40 + 40	Le Fort I	Long-term	1–10 years	Anterior dentition	Not reported	Periodontal exam	Not reported	Clinical recession & CAL	Not reported	No clinically significant long-term GR	No statistically significant differences	Probing depth, gingival index, keratinized tissue width, recession and attachment level measured; mean periodontal changes ≤0.3 mm
Foushee et al., 1985 [16]	Observational clinical	24 patients	Mandibular orthognathic ± genioplasty	Post-treatment	Pre/post	Mandibular anterior	Yes	Periodontal probing (PCP-8)	Single examiner	CEJ–gingival margin	Periodontal evaluation prior to treatment	GR in 6 patients	Significant reduction in keratinized/attached gingiva; CI not reported	Keratinized gingiva width, attached gingiva, sulcus depth and GR measured pre- and post-treatment; recession clinically significant in 6/24 patients

**Table 2:** Structured heterogeneity assessment of included studies.

Study	Follow-Up Duration	Recession Measurement Method	Baseline Periodontal Status Reported	Orthodontic Mechanics Described	Adjunctive Therapy	Main Heterogeneity Driver
Liu et al., 2024 [10]	Up to 12 months (short–mid-term)	Clinical periodontal probing; gingival thickness and keratinized tissue assessment	Yes	Yes (presurgical decompensation)	PAOO vs. non-PAOO	Adjunctive periodontal therapy; phenotype variability
Saab et al., 2023 [11]	Immediate post-treatment (short-term)	Clinical recession assessment of mandibular incisors	Not reported	Yes (compensatory vs. surgical orthodontics)	None	Treatment modality comparison; short follow-up
Weinspach et al., 2011 [12]	6 weeks (short-term)	Periodontal probing; buccal recession measurements	Yes	Limited description	None	Short-term surgical tissue response
Weinspach et al., 2011 [13]	6 weeks (short-term)	Periodontal and microbiological assessment; probing depth and GR	Yes	Limited description	None	Early postoperative tissue changes
Ari-Demirkaya et al., 2008 [14]	6–12 months (mid-term)	Clinical periodontal measurements of mandibular incisors	Yes	Yes (orthodontic decompensation/relapse forces)	None	Orthodontic biomechanics; relapse forces
Carroll et al., 1992 [15]	1–10 years (long-term)	Periodontal examination; recession and attachment levels	Not reported	Yes	None	Long-term periodontal adaptation
Foushee et al., 1985 [16]	6–12 months (mid-term)	Clinical mucogingival assessment; recession prevalence	Not reported	Limited description	None	Baseline mucogingival variability

**Table 3:** Mandibular incisor recession outcomes.

Study	Recession Outcome
Liu et al. (2024) [10]	Significantly higher recession in the NS compared to the PAOO group post-treatment
Saab et al. (2023) [11]	No significant difference between the compensatory and surgical groups
Weinspach et al. (2011) [13]	Small but statistically significant buccal recession post-surgery
Ari-Demirkaya et al. (2008) [14]	No significant effect of relapse on recession
Carroll et al. (1992) [15]	No clinically significant increase long term
Foushee et al. (1985) [16]	Clinically significant recession in subset

**Table 4:** Newcastle–Ottawa scale assessment for the included studies.

Study	Selection Bias	Comparability	Outcome Assessment	Follow-Up Adequacy	Overall Risk of Bias
Liu et al., 2024 [10]	Low	Low	Low	Low	Low
Saab et al., 2023 [11]	Moderate	Moderate	Moderate	Not reported	Moderate
Weinspach et al., 2011 [12]	Moderate	Moderate	Moderate	Short-term only	Moderate
Weinspach et al., 2011 [13]	Moderate	Moderate	Moderate	Short-term only	Moderate
Ari-Demirkaya et al., 2008 [14]	Moderate	Moderate	Moderate	Adequate	Moderate
Carroll et al., 1992 [15]	Moderate	Moderate	Moderate	Long-term	Moderate
Foushee et al., 1985 [16]	High	Moderate	Moderate	Limited	High

**Table 5:** Summary of gingival recession outcomes.

Study	Surgical Procedure	Follow-Up Duration	Tooth Region	Direction of Gingival Recession	Magnitude of Recession	Timing of Detection	Key Risk Modifier Identified
Liu et al., 2024 [10]	Bimaxillary ± PAOO	≤12 months	Mandibular incisors	↑ Increase (non-PAOO); ↔ Stable (PAOO)	Clinically relevant	Post-treatment	Thin phenotype; absence of PAOO
Saab et al., 2023 [11]	Orthognathic vs. compensatory	Immediate	Mandibular incisors	↔ No significant change	Not reported	Immediate post-treatment	None identified
Weinspach et al., 2011 [12]	BSSO ± Le Fort I	6 weeks	Buccal surfaces	↑ Increase	~0.2 mm (mean)	Early postoperative	Surgical site proximity
Weinspach et al., 2011 [13]	BSSO ± Le Fort I	6 weeks	Buccal surfaces	↑ Increase	Small, statistically significant	Early postoperative	Surgical trauma (transient)
Ari-Demirkaya et al., 2008 [14]	Mandibular setback	6–12 months	Mandibular incisors	↔ No significant change	Not clinically significant	Post-treatment	None identified
Carroll et al., 1992 [15]	Le Fort I ± orthodontics	1–10 years	Anterior teeth	↔ Stable	Clinically insignificant	Long-term	None identified
Foushee et al., 1985 [16]	Mandibular orthognathic surgery	6–12 months	Mandibular anterior region	↑ Increase (subset)	Clinically significant in 6/24	Post-treatment	Reduced keratinized gingiva

## Data Availability

The following supporting data are available from the corresponding author upon request.

## Supplementary Materials

The following supporting information can be downloaded at: https://www.mdpi.com/article/10.3390/jcm15051793/s1, Table S1: Detailed electronic search strategies; Table S2: Extracted dataset of study characteristics and periodontal outcomes; Table S3: Newcastle–Ottawa Scale (NOS) risk-of-bias assessment; File S1. PRISMA 2020 checklist.

## References

[1] Harrington C., Gallagher J.R., Borzabadi-Farahani A. (2015). A retrospective analysis of dentofacial deformities and orthognathic surgeries using the index of orthognathic functional treatment need (IOFTN). Int. J. Pediatr. Otorhinolaryngol..

[2] Liu J.Y., Li G.F., Tang Y., Yan F.H., Tan B.C. (2022). Multi-disciplinary treatment of maxillofacial skeletal deformities by orthognathic surgery combined with periodontal phenotype modification: A case report. World J. Clin. Cases.

[3] Mota de Paulo J.P., Herbert de Oliveira Mendes F., Gonçalves Filho R.T., Marçal F.F. (2020). Combined Orthodontic-Orthognathic Approach for Dentofacial Deformities as a Risk Factor for Gingival Recession: A Systematic Review. J. Oral Maxillofac. Surg..

[4] Jepsen S., Caton J.G., Albandar J.M., Bissada N.F., Bouchard P., Cortellini P., Demirel K., de Sanctis M., Ercoli C., Fan J. (2018). Periodontal manifestations of systemic diseases and developmental and acquired conditions: Consensus report of workgroup 3 of the 2017 World Workshop on the Classification of Periodontal and Peri-Implant Diseases and Conditions. J. Periodontol..

[5] Te Veldhuis E.C., Te Veldhuis A.H., Bramer W.M., Wolvius E.B., Koudstaal M.J. (2017). The effect of orthognathic surgery on the temporomandibular joint and oral function: A systematic review. Int. J. Oral Maxillofac. Surg..

[6] Junior O.L.H., Guijarro-Martínez R., Gil A.P.S., Meirelles L.S., Oliveira R.B., Hernández-Alfaro F. (2017). Stability and surgical complications in segmental Le Fort I osteotomy: A systematic review. Int. J. Oral Maxillofac. Surg..

[7] Alsino H.I., Kheshfeh M.N., Hajeer M.Y., Burhan A.S., Alkhouri I., Al-Ibrahim H.M., Abou Nassar J.N., Alsino H.I., Burhan A.S. (2024). Dental and Periodontal Changes After Accelerated Correction of Lower Anterior Crowding With a PAOO Procedure: A Randomized Controlled Trial. Cureus.

[8] Kao R.T., Curtis D.A., Kim D.M., Lin G.H., Wang C.W., Cobb C.M., Hsu Y.T., Kan J., Velasquez D., Avila-Ortiz G. (2020). American Academy of Periodontology best evidence consensus statement on modifying periodontal phenotype in preparation for orthodontic and restorative treatment. J. Periodontol..

[9] Tepedino M., Franchi L., Fabbro O., Chimenti C. (2018). Post-orthodontic lower incisor inclination and gingival recession—A systematic review. Prog. Orthod..

[10] Liu J., Xu X., Yang H.F., Han Y., Pan M.Q., Xu L., Hou J.X., Li X.T. (2024). A nomogram prediction of gingival recession in mandibular incisors of orthodontic-orthognathic treated skeletal class III malocclusion with or without PAOO: A retrospective cohort study. Heliyon.

[11] Saab F.J., de Freitas D.S., Cotrin P., Oliveira R.C., Valarelli F.P., de Oliveira R.C., Salmeron S., Vercelino C.R., Freitas K.M. (2023). Comparison of gingival recession of mandibular incisors of Class III patients immediately after compensatory or surgical orthodontic treatment. Eur. J. Dent..

[12] Weinspach K., Staufenbiel I., Günay H., Geurtsen W., Schwestka-Polly R., Demling A.P. (2011). Influence of orthognathic surgery on periodontal tissues. Int. J. Periodontics Restor. Dent..

[13] Weinspach K., Demling A., Günay H., Geurtsen W., Schwestka-Polly R., Staufenbiel I. (2011). Short-term periodontal and microbiological changes following orthognathic surgery. J. Orofac. Orthop..

[14] Ari-Demirkaya A., Ilhan I. (2008). Effects of relapse forces on periodontal status of mandibular incisors following orthognathic surgery. J. Periodontol..

[15] Carroll W.J., Haug R.H., Bissada N.F., Goldberg J., Hans M.G. (1992). The effects of the Le Fort I osteotomy on the periodontium. J. Oral Maxillofac. Surg..

[16] Foushee D.G., Moriarty J.D., Simpson D.M. (1985). Effects of mandibular orthognathic treatment on mucogingival tissues. J. Periodontol..

[17] Al-Jewair T. (2021). Orthodontic–orthognathic surgical treatment may not be a major risk factor for gingival recession. J. Evid.-Based Dent. Pract..

[18] Demirsoy K.K., Türker G., Amuk M., Kurt G. (2022). How much should incisors be decompensated? Periodontal bone defects during presurgical orthodontic treatment in Class III double-jaw orthognathic surgery patients. J. Stomatol. Oral Maxillofac. Surg..

[19] Halimi A., Zaoui F. (2013). Surgical–orthodontic treatment of patients suffering from severe periodontal disorders—A clinical case study. Int. Orthod..

[20] Posnick J.C., Fantuzzo J.J., Troost T. (2016). Segmental Maxillary Osteotomies in Conjunction with Bimaxillary Orthognathic Surgery: Indications—Safety—Outcome. J. Oral Maxillofac. Surg..

[21] Mirabella D., Macca U., Pancari C., Giunta G., Lombardo L. (2022). Detailed three-dimensional orthodontic tooth repositioning to improve restorative outcome. Angle Orthod..

[22] Idris M.I., Burhan A.S., Hajeer M.Y., Sultan K., Nawaya F.R. (2024). Efficacy of the injectable platelet-rich fibrin (i-PRF) in gingival phenotype modification: A systematic review and meta-analysis of randomized controlled trials. BMC Oral Health.

[23] Wennström J.L. (1996). Mucogingival considerations in orthodontic treatment. Semin. Orthod..

[24] Proffit W.R., Fields H.W., Sarver D.M. (2013). Contemporary Orthodontics.

[25] Lang N.P., Löe H. (1972). The relationship between the width of keratinized gingiva and gingival health. J. Periodontol..

[26] Maynard J.G., Wilson R.D. (1980). Diagnosis and management of mucogingival problems in children. Dent. Clin. N. Am..

[27] Wilcko W.M., Wilcko T., Bouquot J.E., Ferguson D.J. (2001). Rapid orthodontics with alveolar reshaping: Two case reports of decrowding. Int. J. Periodontics Restorative Dent..

[28] Wilcko M.T., Wilcko W.M., Pulver J.J., Bissada N.F., Bouquot J.E. (2009). Accelerated osteogenic orthodontics technique: A 1-stage surgically facilitated rapid orthodontic technique with alveolar augmentation. J. Oral Maxillofac. Surg..

