# Supplementary material for: Gingival Recession After Combined Orthodontic–Orthognathic Treatment: A Systematic Review of Clinical Studies with Emphasis on Mandibular Incisors and Adjunctive Periodontal Therapies
Source: J Clin Med. 2026 Feb 27;15(5):1793. doi: 10.3390/jcm15051793 (PMC12986445; doi:10.3390/jcm15051793)
Supplement: Supplementary file 1 [file jcm-15-01793-s001.zip › jcm-4148003 Supplementary/jcm-4148003 Supplementary 2.docx]

# Supplementary Material S1

Extracted dataset of study characteristics and periodontal outcomes

| Study | Sample | Surgery | Tooth region | Follow-up | GR measurement | Baseline periodontal status | GR outcome | Periodontal parameters | Adjunctive therapy |
| --- | --- | --- | --- | --- | --- | --- | --- | --- | --- |
| Liu 2024 | 33 pts | Bimaxillary ± PAOO | Mandibular incisors | ≤12 mo | Probing | Reported | ↑ GR non‑PAOO | Gingival thickness, KT width | PAOO |
| Saab 2023 | 40 pts | Surgical vs compensatory | Mandibular incisors | Immediate | Photographic | Not reported | No difference | GR depth (mm) | None |
| Weinspach 2011a | 15 pts | BSSO ± Le Fort I | Buccal/oral | 6 weeks | Probing | Reported | ↑ buccal GR | PPD, CAL | None |
| Weinspach 2011b | 15 pts | BSSO ± Le Fort I | Buccal/oral | 6 weeks | Probing + microbiology | Reported | ↑ buccal GR | GR, PPD, plaque index | None |
| Ari-Demirkaya 2008 | 36 pts | Mandibular setback | Mandibular incisors | 6–12 mo | Clinical + radiographic | Reported | No significant GR | Sulcus depth, bone height | None |
| Carroll 1992 | 40 + 40 | Le Fort I | Anterior teeth | 1–10 yrs | Periodontal exam | Not reported | Stable | PD, CAL, KT width | None |
| Foushee 1985 | 24 pts | Mandibular orthognathic | Mandibular anterior | 6–12 mo | Probing | Reported | GR subset | KT width, attached gingiva | None |

# Supplementary Material S2

Newcastle–Ottawa Scale (NOS) risk-of-bias assessment

| Study | Selection | Comparability | Outcome | Total / Risk |
| --- | --- | --- | --- | --- |
| Liu 2024 | ★★★★ | ★★ | ★★★ | 9 / Low |
| Saab 2023 | ★★★ | ★ | ★★ | 6 / Moderate |
| Weinspach 2011a | ★★★ | ★ | ★★ | 6 / Moderate |
| Weinspach 2011b | ★★★ | ★ | ★★ | 6 / Moderate |
| Ari-Demirkaya 2008 | ★★★ | ★★ | ★★ | 7 / Moderate |
| Carroll 1992 | ★★★ | ★★ | ★★ | 7 / Moderate |
| Foushee 1985 | ★★ | ★ | ★★ | 5 / High |

# Supplementary Material S3

Detailed electronic search strategies

## PubMed/MEDLINE

("gingival recession"[MeSH] OR "periodontal recession" OR "gingival margin") AND ("orthognathic surgery"[MeSH] OR "jaw surgery" OR "Le Fort I" OR "sagittal split osteotomy") AND ("orthodontic treatment" OR "combined orthodontic orthognathic")

## Web of Science

TS=(gingival recession OR periodontal recession OR gingival margin) AND TS=(orthognathic surgery OR Le Fort OR BSSO OR jaw surgery) AND TS=(orthodontic)

## Cochrane Library

(gingival recession OR periodontal recession) AND (orthognathic surgery OR maxillary osteotomy OR mandibular osteotomy)

## Manual search

Reference lists of included studies and relevant reviews were screened.
